# Association of Single Nucleotide Polymorphisms in the IL-18 Gene with Production of IL-18 Protein by Mononuclear Cells from Healthy Donors

**DOI:** 10.1155/2008/309721

**Published:** 2008-10-20

**Authors:** O. P. Khripko, N. S. Sennikova, J. A. Lopatnikova, J. I. Khripko, M. L. Filipenko, E. A. Khrapov, E. L. Gelfgat, E. V. Yakushenko, V. A. Kozlov, S. V. Sennikov

**Affiliations:** ^1^Laboratory of Molecular Immunology, Institute of Clinical Immunology , Siberian Branch of Russian Academy of Medical Sciences (SB RAMS), Novosibirsk 630099, Russia; ^2^Pharmacogenomic Group, Institute of Chemical Biology and Fundamental Medicine, Novosibirsk 630090, Russia

## Abstract

IL-18 has proinflammatory effects and participates in both innate and adaptive cellular and humoral immunity. A number of SNPs that influence IL-18 production are found in the gene promoter region. We investigated the association of SNPs in the IL-18 promoter at *−*607 and *−*137 with the level of IL-18 protein production by PBMC from healthy donors from Southwestern Siberia. The genetic distribution of these SNPs in the promoter site was established by PCR. IL-18 protein production was determined by ELISA. Our results showed that PBMC from donors carrying allele 137C have lower levels of both spontaneous and LPS-stimulated IL-18 production. In contrast, PBMC from donors carrying allele 607A showed significant increases in spontaneous and stimulated IL-18 production compared to wild type. Our study suggests that the SNPs *−*607 and *−*137 in the promoter region of the IL-18 gene influence the level of IL-18 protein production by PBMC from healthy donors in Southwestern Siberia.

## 1. INTRODUCTION

IL-18 is a pleiotropic proinflammatory cytokine that stimulates production of IFN-*γ*, TNF-*α*, IL-1, IL-2, adhesion molecules and apoptosis factors, increasing T-lymphocyte proliferation, and enhancing the lytic activity of NK-cells. It participates in the cellular and humoral immune response [1], both innate and adaptive [2]. It has been shown that IL-18 is involved in the pathogenesis of various diseases including type-I diabetes [3], rheumatoid arthritis [4], Crohn's disease [5], and liver cirrhosis [6]. Allelic variants of cytokine genes associated with promoter gene region polymorphisms do not influence the protein amino acid sequence but can result in changes in cytokine production. In consequence, they may alter the immune responses mediated by the cytokine in question and could be associated with various immunological diseases. Three single nucleotide polymorphisms (SNPs) are found in the IL-18 gene promoter in positions −656 G*→*T, −607 C*→*A, and −137 G*→*C. Two of these, −607 C*→*A and −137 G*→*C, are located at the binding sites for CREB transcriptional factors (cAMP response-element binding proteins) and the H4TF-1 nuclear factor, respectively, therefore mutation at these two sites could influence IL-18 expression and change the production of the cytokine [7]. Recent studies have investigated the relationship between IL-18 gene polymorphisms and predispositions to various diseases. It has been shown that individuals carrying the −607C −137C haplotype are prevalent in rheumatoid arthritis cases among the populations of Germany and Scotland. Women carrying the C-allele at −137 have a heightened risk of developing ovarian carcinoma [8]. The presence of such a genotype in patients with nasopharyngeal carcinoma of an undifferentiated type worsens the prognosis for the disease and is associated with earlier metastasis [9]. The G-allele at −137 is associated with the development of bronchial asthma and dermal allergic reactions [10]. In patients with hepatitis B from the Chinese population, the −137GG and −607CA allele variants of the IL-18 gene promoter are consistently more frequent; conversely, hepatitis B is encountered consistently more rarely in persons with the C-allele at −137 [11]. Nevertheless, the impact of one or other IL-18 allele variant on the level of protein production has not been adequately studied.

The aim of our study was therefore as follows: to investigate the distribution of IL-18 allele variants at positions −607C*→*A and −137G*→*C within a population of healthy donors from Southwestern Siberia, and the influence of these allele variants on the level of IL-18 production by their peripheral blood mononuclear cells (MNCs).

## 2. MATERIALS AND METHODS

### 2.1. Object of research

DNA was obtained from PBMC from adult (20–50 years of age) healthy donors (*n* = 146) to study polymorphisms in the promoter region of the IL-18 gene and the production IL-18 protein. All subjects gave written informed consent for enrolment in the study, which was approved by the local Ethics Committee.

### 2.2. Definition of genotypes IL-18

Genomic DNA was extracted from PBMC by a set Test-NA (DNA-technology, Russia). Allele-specific amplification was assessed by fluorescent detection of the products of real-time PCR. Four primers were used for amplification, two specific for each polymorphism at positions −607 and −137. Amplification was controled by paired primers specific to the particular genomic site. Products were detected using the intercalating dye SYBR-green I. PCR was performed using an iQ4 Cycler (Bio-Rad, USA). The reaction mixture (15 *μ*l final volume) contained 0.3 *μ*M specific and control primers; PCR buffer (16 mM Tris-HCl, pH 8.9, 2.4 mM MgCl_2_, 65 mM (NH_4_)_2_SO_4_); 0.2 mM dNTP; 1× SYBR-green I; 1 unit Taq-polymerase; and 5 ng DNA. The PCR conditions were as follows: denaturation (95°C, 3 minutes) followed by 5 cycles of amplification at 95°C for 10 seconds, 57°C for 10 seconds, and 72°C for 10 seconds; then 30 cycles at 95°C for 3 seconds, 59°C for 3 seconds, 72°C for 3 seconds, 78°C for 10 seconds (plate read), and 82°C for 10 seconds (plate read). A melting curve analysis was then performed: 60 cycles starting at 65°C with temperature increments of 0.5°C, the plate being read at each cycle. The data obtained were interpreted on the basis with the increase in fluorescence, and the melting curve was used to assess specificity. The specific primers used were

137 C 5′-TAATGTAATATCACTATTTTCATGAAATC-3′
137 G 5′-AATGTAATATCACTATTTTCATGAAATG-3′
607 C 5′-GTTGCAGAAAGTGTAAAAATTATTAC-3′
607 A 5′-GTTGCAGAAAGTGTAAAAATTATTAA-3′.

The internal control primers were

LTM 1 tgggtgctagaggtataatcg
LTM 2 ttagaggaagctgggtaagag.

As we used two pairs of allele-specific primers, we studied the reactions in four test tubes simultaneously for each sample to detect any of the four possible haplotypes.

### 2.3. Preparation of conditioned medium from PBMC

PBMC were isolated from heparinized blood using Ficoll-Urografin density gradients and were cultured (1 × 10^6^ cells/mL) for 48 hours in a 48-well plate (Beckton Dikkinson, USA) in the presence or absence of LPS (055/B5, Sigma, USA) at a final concentration of 10 *μ*g/mL.

### 2.4. Definition of IL-18 level

The level of IL-18 protein in the conditioned medium was determined by ELISA (Bender Med Systems, Austria).

### 2.5. Statistical analysis

All SNPs were in Hardy-Weinberg equilibrium in investigated population.For statistical analysis of the data, we used the nonparametric Mann-Uitni's test employing the Statistica 6.0 program. To identify quantitative differences in IL-18 production levels, we used quantile rank classification according to the Mosteller-Tewkee algorithm [12]. To analyze the IL-18 concentrations, we determined the probability limits of the empirical distribution quantiles—(X(qi)). A number of quantile ranges (K), into which the total amplitude of variation of the IL-18 level was split, were calculated starting from a minimal sample size (Nmin) by Sturges's formula. *χ* ^2^-criterion (P*χ*) and Fisher's precise method for small samples (Ptmf) were used to judge the reliability ofoccurrence for qualitative features and frequencies of events within different ranges of IL-18 variation [13].

## 3. RESULTS

### 3.1. IL-18 gene promoter genotype distribution

The distribution of the susceptibility/resistance gene alleles responsible for resistance to or risk of developing immunopathological diseases is unique to each population. This could be one reason for the diversity of immune responses to an antigen. This study of two SNPs in the IL-18 gene was conducted on a group of healthy persons (*n* = 146). The relative frequencies of the C/C, C/A, and A/A variants in the 607C*→*A polymorphism were 39.2%, 44.7%, and 16.1%, respectively. The C and A alleles occurred at frequencies of 64.5% and 35.5%, respectively. Analysis of the IL-18 gene polymorphism at the −137*→*G position gave the following results: the frequency of the homozygous variant (G/G) was 45.5%, and that of the heterozygous variant G/C was 42.7%; the C/C genotype was found in 11.8% of cases, and the G and C allele frequencies were 66.8% and 33.2%, respectively. The frequencies of the AC ( −607/−137) haplotype were 22%, AG 14.6%, CG 55.9%, and CC 7.5%.

### 3.2. IL-18 production related to the −607C *→* A polymorphism

We next investigated the IL-18 concentration in media conditioned by the PBMC from healthy donors. The median levels of IL-18 protein were 21.86 pg/mL [9.83; 57.60 (quartiles)] for spontaneous production and 72.44 pg/mL [32.96; 103.07 (quartiles)] for LPS-stimulated production. We further studied the correlation between IL-18 production and the polymorphic variants −607C*→*A in the IL-18 gene promoter (see Figure 2).

IL-18 production by LPS-stimulated PBMC was significantly greater in healthy donors carrying the CA genotype than in those with the CC genotype at the −607 position of the promoter. We analyzed the relationship between genotype frequency distributions and IL-18 production, dividing the cytokine concentrations into “low,” “medium,” and “high” classes. Concentrations in the 15–85% quantile range (3.5–93.5 pg/mL) for spontaneous production were classed as “medium.” Production levels outside these limits were classed as “low” or “high” (see Table 1). We found a higher frequency of A-allele carriers among persons with a high level of spontaneous IL-18 production.

### 3.3. IL-18 production related to the −137G *→* C polymorphism

**Figure 1:**
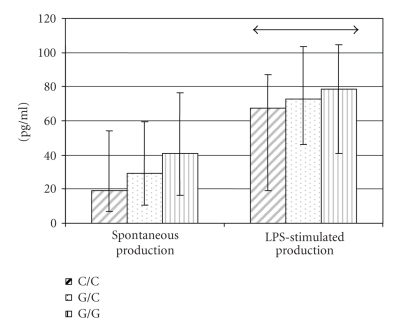
Effect of polymorphic variants in the IL-18 gene promoter region at −137 G*→*C on IL-18 protein production by PBMC (data are represented as median and 25 and 75 percentiles) (↔p_Mann-Uitni_ = 0.0494).

**Figure 2:**
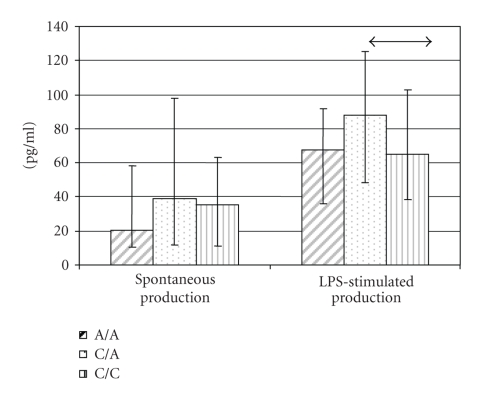
Effect of polymorphic variants in the promoter region of the IL-18 gene at the −607 A*→*C position on IL-18 production by PBMC (data are represented as median and 25 and 75 percentiles) (↔p_Mann-Uitni_ = 0.045).

We studied the correlation between IL-18 production by PBMC from healthy donors and variants of the IL-18 genotype −137 at the position, and we obtained the following data (see Figure 1).

IL-18 production by LPS-stimulated PBMC was significantly less in persons carrying the CC genotype than in those with the GG genotype at the −137 position. To investigate this further, we analyzed the relationship between the genotype frequency distribution and levels of IL-18 production. The limits of three primary ranges of variation in IL-18 production levels were determined by quantile rank analysis which is tolerant of random statistical “surges” and critical deviations of a sign from normal distribution. In accordance with common practice, all empirically obtained concentrations of this cytokine were classified as “low,” “medium,” or “high.” Concentrations in the range 15–85% (25.6–114.3 pg/mL) for LPS-stimulated production were classed as “medium.” Concentrations outside the limits of these quantiles, that is, less than 25.6 pg/mL and more than 114.3 pg/mL, were classed as “low” and “high,” respectively.

We evaluated the allele variant frequencies among producers of different levels of IL-18. The results are shown in Table 2.

The frequency of the C allele at the −137 position was significantly higher in donors with low LPS-stimulated IL-18 production than in those with medium or high production. Thus, LPS-stimulated production of IL-18 is lower in persons with the CC genotype than in donors with the GG genotype at the −137 position of the promoter. The C allele frequency is greater in the group with a low level of stimulated IL-18 production.

### 3.4. Capacity to produce IL-18 by mononuclear cells is associated with IL-18 polymorphism

**Figure 3:**
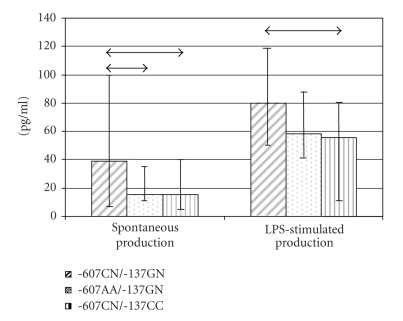
Capacity to produce IL-18 by mononuclear cells is associated with −607/−137 IL-18 polymorphism (data are represented as median and 25 and 75 percentiles) (↔p_Mann-Uitni_ < 0.05).

As these two polymorphisms, −137 G/C and −607 C/A, were in strong linkage disequilibrium [14], all possible combinations of alleles −607 C*→*A and −137 G*→*C have been investigated. There has been shown increased spontaneous production of IL-18 associated with haplogenotypes −607CN/−137GN in comparison with −607AA/−137GN and −607CN/−137CC. Thus alleles −607C and −137G are associated with increase of spontaneous production of IL-18. Increased LPS-stimulated production of IL-18 is associated with a haplogenotype −607CN/−137GN in comparison with −607CN/−137CC (see Figure 3).

This study showed that the frequencies of IL-18 genotypes in healthy donors from Novosibirsk city (Southwestern Siberia) were as follows: −607CC, 32.2%; −607AC, 44.7%; −607AA, 16.1%; −137GG, 45.5%; −137GC, 42.7%; and −137CC, 11.8%. For the haplotypes, the values were as follows: AC (−607/−137), 22%; AG, 14.6%; CG, 55.9%; and CC, 7.5%. LPS-stimulated production of IL-18 was lower in persons with the CC genotype than in donors with the GG genotype at the −137 position of the gene promoter. IL-18 production by LPS-stimulated PBMC from healthy donors carrying the CA genotype was significantly higher than from those carrying the CC genotype at the −607 position. Alleles −607C and −137G are associated with increase of spontaneous production of IL-18, allele −137G increases spontaneous and stimulated production.

## 4. DISCUSSION

Genetic factors are important in the pathogenesis of all major human diseases and it is known that human populations vary genetically because of polymorphisms, which can affect aspects of the immune response. Diverse polymorphic variants of cytokine genes may induce high or low levels of protein production. Single nucleotide polymorphisms in the IL-18 gene promoter at −607 C*→*A and −137 G*→*C affect functionally active parts of this promoter, that is, binding sites for CREB transcriptional factors and the H4TF-1 nuclear factor, respectively. Therefore, mutation at these two sites could influence IL-18 expression and alter the level of IL-18 production [7].

Considering the distributions of IL-18 gene alleles in healthy donors from different populations, we found various frequency rates. In the Japanese population, the genotypes GG, GC, and CC were found at the −137 position of the promoter with frequencies of 75.4%, 23.1%, and 1.2%, respectively; the genotypes AA, AC, and CC at the −607 position had the frequencies 43.9%, 39.2%, and 16.9%, respectively [15]. Genotype frequencies in the Chinese population were −137GG, 67.3%; −137GC, 30%; −137CC, 2.7%; −607AA, 24.7%; −607AC, 53.3%; and −607CC, 22% [11]. For European populations from Switzerland [10] and Italy [9], the characteristic frequencies of the genotypes −137GG, −137GC, and −137CC were 54.1% and 48.3%, 38.7% and 43.8%, and 7.2% and 7.9%, respectively. IL-18 genotypes at the −607 position of the promoter were found in the Italian population at the following rates: AA, 23.6%; AC, 47.2%; CC, 29.2% [9].

We found the following IL-18 genotype frequencies in healthy donors from Novosibirsk (Southwestern Siberia): GG, GC, and CC at the −137 position of the promoter were found in 45.5%, 42.7%, and 11.8% of cases, respectively; AA, AC, and CC at the −607 position were found in 16.1%, 44.7%, and 39.2%, respectively. Thus, the genotype frequencies within the population of Southwestern Siberia are similar to those of European countries (Italy and Switzerland). There are, however, some differences in genotype distribution between the inhabitants of Southwestern Siberia and the populations of China and Japan.

It is well known that gene promoter polymorphisms can affect the level of protein production. Our study demonstrated a relationship between two IL-18 gene promoter polymorphisms and the level of LPS-stimulated IL-18 production. PBMC from persons carrying the C allele at the −137 position of the promoter show low LPS-stimulated production of the cytokine. Also, the level of IL-18 production by PBMC from donors with the −607AC genotype is higher than that from donors with the −607CC genotype. The A allele is likewise associated with greater LPS-stimulated IL-18 production. There appears little dose effect of the allele A of −607 SNP and we could not detect any difference of IL-18 production when heterozygote for −137 SNP was considered as a whole. However, after splitting heterozygote for one SNP according to their genotype at other SNP, we were able to demonstrate differential haplogenotypic IL-18 production. The −607CN/−137GN haplogenotype was associated with a higher spontaneous IL-18 production. Increased LPS-stimulated production of IL-18 was associated with a haplogenotype −607CN/−137GN in comparison with −607CN/−137CC. Thus alleles −607C and −137G are associated with increase of spontaneous production of IL-18, allele −137G increases spontaneous and stimulated production. It is possible to assume that only in case of presence of both alleles conducts to increase production of IL-18 protein. The increased frequency of allele −607A with high production IL-18 is connected with heterozygotes −607CA (16 donors from 19).

Arimitsu et al. [16] showed that a polymorphic variant of the IL-18 gene influenced IL-18 production by monocytes. In particular, they showed that spontaneous and LPS-stimulated production of IL-18 in volunteers with the −137GG genotype was higher than in those with the −137GC genotype, which is in agreement with our results.

IL-18 is a pleiotropic proinflammatory cytokine that predominantly influences the differentiation of type 1 T-helper cells, thus participating in the establishment of the cellular immune response and inflammatory reactions. IL-18 has been shown to have antitumor activity. A study of the relationship between IL-18 gene promoter polymorphisms and the likelihood of developing oncological diseases showed that women carrying the C allele in the −137 position of the promoter are at greater risk of developing ovarian carcinoma [8]. On the basis of our results and data from the literature, one could suppose that the presence of the C allele at the −137 position in the IL-18 gene promoter in such patients results in a less efficient antitumor immune response because IL-18 production by immunocompetent cells is diminished.

The serum level of the proinflammatory factor IL-18 is known to be significantly elevated in patients with atopic asthma during the acute phase [17]. Studies of the association between IL-18 polymorphism and atopic asthma have revealed that the G allele at the −137 position leads to an elevated risk of disease development; moreover, none of the 74 patients examined had the CC genotype [10], which according to our data is associated with reduced IL-18 production. Moreover, the linkage disequilibrium was observed between the −137 and 105 polymorphisms of the IL-18 gene and the 105A allele of the IL-18 gene which may be associated with the pathogenesis of asthma [18].

Premature infants with reduced birth weight are at risk of developing necrotizing enterocolitis and IL-18 that is also involved in the pathogenesis of this disease. Analysis of the association between disease severity and genotype frequencies at the −607 position of the IL-18 gene showed that the AA genotype frequency is significantly higher in patients with the third and most severe stage of necrotizing enterocolitis, when the intestinal wall is perforated [19].

On the basis of present knowledge, one may assume that allelic variants of the IL-18 gene promoter at the −607C*→*A and −137G*→*C positions, which, according to our results, influence the level of protein production, could result in conditions of which the pathogenesis involves a significant role for IL-18.

In conclusion, this study shows that the IL-18 genotype frequencies among healthy donors from Novosibirsk city (Southwestern Siberia) are as follows: −607CC, 39.2%; −607AC, 44.7%; −607AA, 16.1%; −137GG, 45.5%; −137GC, 42.7%; and −137CC, 11.8%. This is consistent with the distributions within European populations (Italy and Switzerland). Allelic variants in the IL-18 gene promoter at the −607C*→*A and −137G*→*C positions influence the level of production of the mediator by immunocompetent cells. LPS-stimulated production of IL-18 in persons with the CC genotype is lower than that in donors with the GG genotype at the −137 position of the promoter. The C allele frequency is significantly higher in the group with low LPS-stimulated IL-18 production. LPS-stimulated production of IL-18 by PBMC from healthy donors is significantly greater in those carrying CA genotype at the −607 position. Thus alleles −607C and −137G are associated with the increase of spontaneous production of IL-18 and allele −137G increases spontaneous and stimulated production. It is possible to assume that only in case of presence of both alleles conducts to increase production of IL-18 protein.

## Figures and Tables

**Table 1:** Distribution of frequencies of IL-18 gene promoter genotypes at the −607 A*→*C position in relation to the level of spontaneous IL-18 secretion.

Quantiles of level of spontaneous	IL-18 level	−607CC	−607AN	(p_emf_)	p*χ* ^2^
IL-18 secretion	pg/mL	*n*	*n*		
Low (0–15%)	0–3.4	7	11	0.2031	>0.1
Medium (15–85%)	3.5–95.3	44	57		
High (85–100%)	95.4–138	5	19	0.0239	<0.05
All		56	87		

**Table 2:** Distribution of frequencies of IL-18 gene promoter genotypes at the −137 G*→*C position in relation to the level of LPS-stimulated IL-18 secretion.

Quantiles of level of LPS-	IL-18 level	−137GG	−137CN	(p_emf_)	p*χ* ^2^
stimulated IL-18 secretion	pg/mL	*n*	*n*		
Low (0–15%)	0–25.6	3	14	0.0098	<0.05
Medium (15–85%)	25.7–114.2	48	50		
High (85–100%)	114.3–298.4	14	14	0.1445	>0.1
All		65	78		
